# A Two-Dimensional Modeling Procedure to Estimate the Loss Equivalent Resistance Including the Saturation Effect

**DOI:** 10.3390/ma6115159

**Published:** 2013-11-12

**Authors:** Rosa Ana Salas

**Affiliations:** Electronic Technology Department, Higher Polytechnic School, Carlos III University of Madrid, Avda. de la Universidad, 30, 28911, Leganés (Madrid), Spain; E-Mail: rsalas@ing.uc3m.es; Tel.: +34-91-624-8864; Fax: +34-91-624-9430

**Keywords:** ferrite cores, finite element analysis (FEA), power losses, nonlinear inductors

## Abstract

We propose a modeling procedure specifically designed for a ferrite inductor excited by a waveform in time domain. We estimate the loss resistance in the core (parameter of the electrical model of the inductor) by means of a Finite Element Method in 2D which leads to significant computational advantages over the 3D model. The methodology is validated for an RM (rectangular modulus) ferrite core working in the linear and the saturation regions. Excellent agreement is found between the experimental data and the computational results.

## 1. Introduction

**Figure 1:**
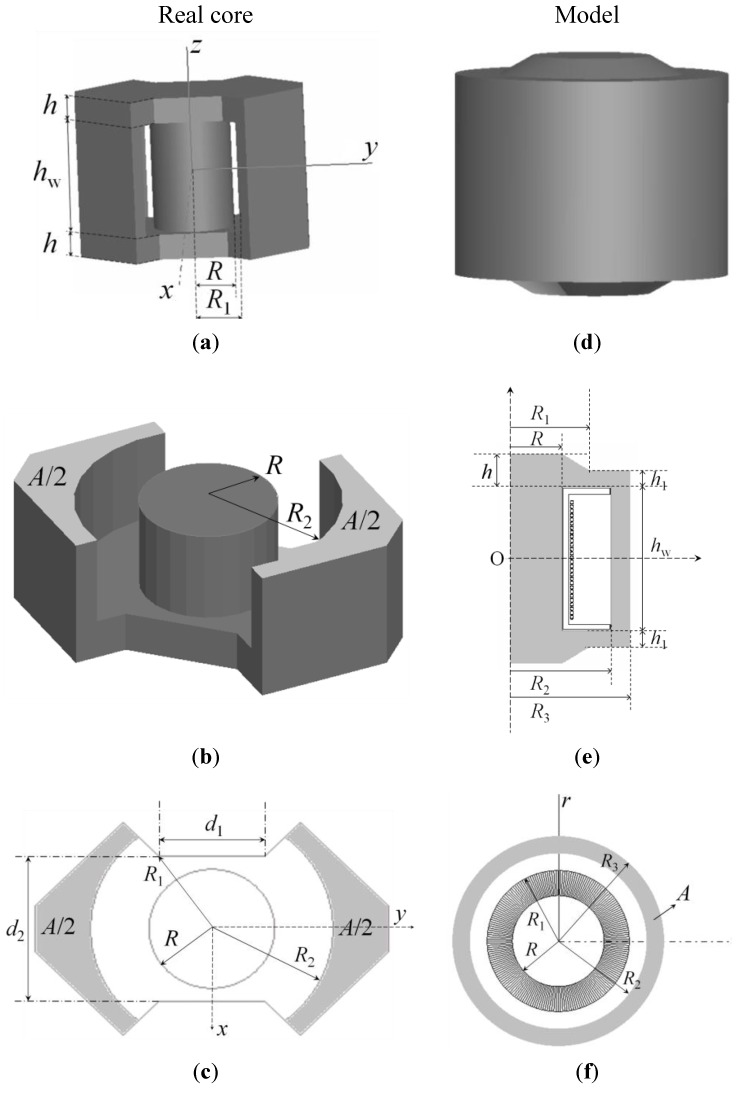
(**a**) RM-shaped real core; (**b**) RM-shaped core half; (**c**) cross-section in the *x*-*y* plane of the RM core; (**d**) solid of revolution; (**e**) 2D equivalent inductor model; and (**f**) cross-section of the solid of revolution.

Soft ferrites are magnetic materials that are commonly recognized as suitable core materials for high-frequency magnetic devices, such as inductors and transformers used in power electronic systems, because of their attractive characteristics of high permeability, low eddy-current loss, and low cost [1,2,3,4,5]. In addition, because of their comparatively low losses at high frequencies, they are extensively used in the cores of RF (radio frequency) transformers and inductors in applications such as switched-mode power supplies [6,7,8]. Nevertheless, ferrites are difficult to model because of their nonlinear behavior (saturation effects and losses) and different geometries. Due to the complex shapes of the cores, as can be seen in the example in Figure 1a, the 3D simulation may not converge or may lead to many hours of computation. Models and linear simulations are found in the literature [9,10,11,12,13,14]. In spite of these studies there is a lack of models that incorporate simultaneously different geometries and sizes and a wide range of working frequencies and regions, including linear, intermediate and saturation regions. They should also allow us to estimate inductance and loss resistance curves to be used in circuit simulators. In previous papers we have proposed a modeling procedure in 2D for the calculation of current dependent inductance in RM (rectangular modulus) and POT ferrite cores without losses [15,16]. The shape of a POT core is round with an internal hollow that almost completely encloses the coil. Usually a POT core is made in two halves which fit together around a coil former.

In this paper, we focus on the calculation of loss resistance in the core and propose a 2D simulation procedure that shows results that are in good agreement with the experimental ones. The procedure is based on the design of a 2D equivalent inductor model for a ferrite RM core (Figure 1a) and is capable of reproducing the voltage and current waveforms when the inductor is excited by a sinusoidal waveform. From these we derive power and resistance curves in the ferrite’s complete working range including the linear and saturation region.

The advantages of our procedure are being able to achieve a reduction of the computational cost in a design and circuit simulator software, incorporate the nonlinear behavior of the ferrite and consider the frequency effect in our simulations.

The structure of the paper is as follows. In Section 2 we present the procedure for calculation of resistance (*R*). In addition we present our 2D equivalent model for the RM core and the design criteria. The results are presented and compared with experimental measurements in Section 3. Finally, in Section 4 we summarize our results and offer some conclusions.

## 2. Materials and Methods

### 2.1. Methodology to Compute R

We begin by specifying the notation that will be used in the rest of this paper. We use **B** and **H** (in bold) to denote the magnetic field vectors. We also use Ф to indicate the magnetic flux; *I* the DC excitation current value that flows through the inductor; and *I*_rms_ is the rms value of the current waveform. The aim of the procedure is to compute *R* as a function of *I*_rms_ (*R*-*I*_rms_ curve). To do this, we propose a procedure that combines experimental measurements with the use of finite element analysis in 2D. The modeling procedure of *R* involves three steps: *premodeling*, *simulation* and *postmodeling*.

At the *premodeling step* we use as input to the simulation program the experimental data of the magnetic behavior of the ferrite: the *B*-*H* curve that characterizes the core material and the values of the Steinmetz coefficients of the ferrite to be analyzed. The physical meaning of these coefficients is described in the literature [17,18,19,20,21,22,23]. While the coefficients account for the power losses, the *B*-*H* curve reproduces the behavior of the material from the linear to the saturation regions. In order to measure these data we follow the methodology described in Section 2.2. Once this is done, we design the new 2D equivalent inductor (including the geometry, winding and coil former, if necessary). Then we define and assign the magnetic properties of the materials such as relative permeability and conductivity of the copper wire, relative permeability of the background, relative permeability and conductivity of the coil former if present, linear conductivity of the ferrite and its *B*-*H* curve. We also define and assign the boundary conditions, the waveform of the excitation voltage (sinusoidal at a fixed frequency and voltage amplitude) and details of the windings such as initial current, resistance *R*_Ω_, leakage inductance, capacitance and number of turns. Finally, we assign the values of the Steinmetz coefficients, define the time parameters to generate the solution (stop time and time step τ), and generate the spatial meshing of the domain.

At the *simulation step* we compute the voltage, current and power waveforms and the instantaneous spatial distribution values of the magnetic fields **B** and **H**. We carry out the simulations by means of the Ansoft Maxwell 2D field simulator’s transient solver. The power losses in the ferrite core are taken into account using the Steinmetz equation [19].

At the *postmodeling step* we compute $\bar{P}$ (average value of the power), *I*_rms_ (rms values of the current waveform) and from these *R*$\left(R=\bar{P}/I_{rms}^{\text{2}}\right)$ from the instantaneous current and power waveforms for each excitation voltage applied to the inductor. These are part of the simulating step’s output.

By repeating the simulation at different excitation voltage values, we obtain the $\bar{P}$-*I*_rms_ curve and from this derive the *R*-*I*_rms_ curve. This calculation can be repeated for each working frequency needed. We have validated the procedure by comparing these results with experimental measurements. 

We have applied our methodology to a MnZn soft ferrite core made of 3F3 material from the manufacturer Ferroxcube. Its main application area is power transformers and inductors as well as general purpose transformers. This material has an initial permeability μ*_i_* = 2000 ± 20% at 25 °C, a saturation flux density *B*_sat_ ≈ 440 mT at 25 °C and *H* = 1200 A/m, Curie temperature *T*_C_ ≥ 200 °C and a DC (direct current) resistivity ρ ≈ 2 Ωm at 25 °C.

### 2.2. Experimental Measurements 

We took two different types of measurements to analyze the nonlinear behavior of the ferrite cores, in order to obtain the input parameters of the 2D FEA (finite element analysis) simulations and to validate the proposed procedure and simulations by comparison with the experimental results. These two different types of measurements are measurements under variable DC current and measurements under variable voltage and frequency.

#### 2.2.1. Measurements under Variable DC Current

The objective of the measurements is to obtain the *B*-*H* curve that characterizes the core material of the ferrite from the linear to the saturation regions. This curve is an input parameter in the simulation program. We derive the *B*-*H* curve from the Ф*-I* curve. For this we build a two-winding transformer with the same number of turns in the primary (*N*_p_) and secondary (*N*_s_) coils. For the transformer we use a toroidal ferrite core with the same material as the inductor to be studied. We apply a variable DC current *I*, generated from a DC power supply, to the primary coil. We measure the magnetic flux Ф in the secondary coil with the Magnet-Physik electronic fluxmeter from the linear to the saturation regions. With these values we compute *B* and *H* as follows:

(1) $$B\approx \frac{1}{N_{s}A_{e}}\int \nu \;dt=\frac{\Phi }{N_{s}A_{e}}$$

(2) $$H\approx \frac{N_{p}I}{\ell_{e}}$$

where *A*_e_ is the effective cross-sectional area of the core; and $\ell_{\text{e}}$ is the effective magnetic path length. These values are defined in the Ferroxcube catalog for each geometry.

#### 2.2.2. Measurements under Variable Voltage and Frequency

The objectives of these measurements are to obtain the Steinmetz coefficients and to validate the output voltage and current waveforms obtained from the proposed procedure to estimate *R*.

For all of this, we measure the voltage ν(*t*) and current *i*(*t*) waveforms by exciting the inductor with a sinusoidal waveform in order to obtain the $\bar{P}$-*I*_rms_ curve from which we derive the *R*-*I*_rms_ curve and the ${\bar{P}}_{\nu }$-*B*_p_ curve from which we derive the Steinmetz coefficients. $\bar{P}$ and ${\bar{P}}_{\nu }$ are respectively the average value of the power and the average value of the power per unit volume of the core. We compute $\bar{P}$ as,

(3) $$\bar{P}=\frac{1}{T}\int_{0}^{T}\nu \left(t\right)\times i\left(t\right)\;dt$$

where *T* is the time period of the waveform. We then compute *R* using the equation $R=\bar{P}/I_{rms}^{2}\cdot$

In order to obtain the values of the Steinmetz coefficients α, β, *C_m_* we fit the experimental data ${\bar{P}}_{\nu }$-*B*_p_ to the Steinmetz equation,

(4) $${\bar{P}}_{\nu }=C_{m}f^{\alpha }B_{p}^{\beta }$$

In this equation *B*_p_ is the maximum value of the magnetic field *B*(*t*) defined as,

(5) $$B\left(t\right)=\frac{1}{NA_{e}}\int \nu \left(t\right)\;dt$$

where *N* is the number of turns of the inductor to be studied.

#### 2.3. 2D Ferrite Core Design

As commented before, our methodology to compute *R* is based on the design of a 2D equivalent inductor. In Figure 1a–c we show an RM core and in Figure 1d–f its 2D model and solid of revolution. The design criteria are based on the need to obtain the same volume and magnetic flux both in the real and the simulated core. In addition, the **B**-field is distributed with the same intensity throughout the two cores. The model is defined by the parameters (*R*, *R*_1_, *R*_2_, *R*_3_, *h*, *h*_1_, *h*_w_) which are easily obtained from the parameters of the real core (*R*, *R*_2_, *h*, *h*_w_, *d*_1_, *d*_2_, *A*), where *A* is the shadowed area in Figure 1c. The parameters *R*, *R*_2_, *h* and *h*_w_ are identical both in the real and modeled core while *R*_1_, *R*_3_ and *h*_1_ have to be calculated from the real core. Thus, *R*_1_ is obtained from *d*_1_ and *d*_2_, *R*_3_ is obtained from *R*_2_ and *A*, and *h*_1_ is derived from *h* as *h*_1_ = *h*/2. As we are able to check in our numerical tests, there is hardly any difference between the real core and its axisymmetric model. This is due to the fact the dimension of the model has been calculated considering that the magnetic flux and volume are the same in both cores. 

## 3. Validation and Results

**Figure 2:**
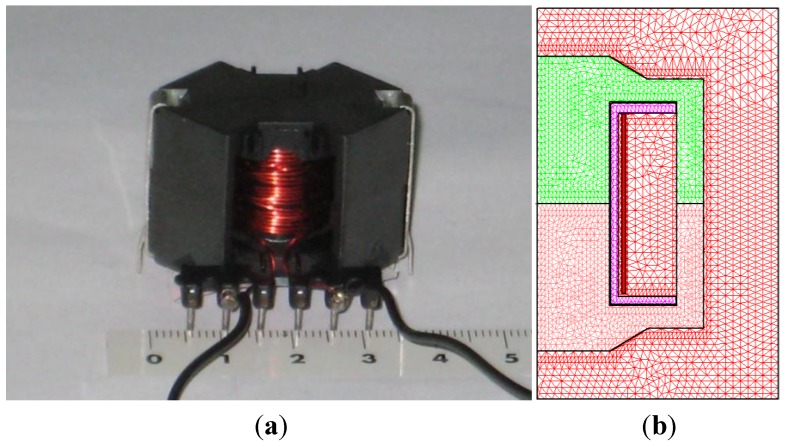
(**a**) Real inductor with an RM-shaped core; and (**b**) 2D model and triangular mesh generated by the 2D simulations.

In order to study said geometry, we use an inductor with an RM core consisting of two identical halves of an RM14/I core with an air-gap length of 7 μm, a coil former and a 28-turn copper coil. In order to make the experimental measurements, we wind another identical copper coil around the first one to build a transformer (Figure 2a). In Figure 2b we can observe the design made for the 2D equivalent inductor.

We carried out our simulations by means of the Ansoft Maxwell 2D field simulator’s transient solver in the *r*-*z* plane obtaining the voltage, current and power waveforms in time domain. We choose *D*: (*r*,*z*) ϵ [0,25] × [−20,20] (unit are millimeters) as a computational domain (Figure 2b) of the 28-turn RM14/I inductor [15]. The dimensions of the 2D inductor model are *R* = 7.5 mm, *R*_1_ = 11.41 mm, *R*_2_ = 14.5 mm, *R*_3_ = 17.24 mm, *h*_w_ = 20.8 mm, *h* = 4.65 mm, *h*_1_ = 2.32 mm. In order to run the simulations we consider both the leakage inductance of the ferrite and the parasitic capacitance to have no effect. For the simulations, we have taken the values of the Steinmetz coefficients for each frequency obtained by experimental measurements. We have also taken the value of the DC resistance of the winding according to *R*_Ω_ = ℓ*/*σ*S* ≈ 0.13 Ω being σ = 5.8 × 10^7^ S∙m^−1^ the copper’s conductivity, *S* the cross-sectional area of the copper wire used and ℓ its length. Note that the winding resistance is more important at low working frequencies. 

The validation of the 2D simulations consists of comparing the simulation results with the experimental measurements obtained with the procedure described in Section 2.2. We carry out the validation of the design of the 2D domain and the analysis of the inductor’s behavior at two frequencies: Low frequency of 500 Hz (quasistatic conditions) and working frequency of the ferrite (40 kHz). As comparing criteria we use the following parameters: Voltage and current waveforms, $\bar{P}$-*I*_rms_ and $\bar{P}$-*I*_rms_ curves and *R*-*I*_rms_ curve, where *V*_rms_ is the rms value of the voltage waveform. We do not show 3D simulation results as we ran simulations and in some cases (saturation) there was no convergence. Thus, we consider that the experimental checking is sufficient.

### 3.1. Results at 500 Hz (Quasistatic Conditions)

**Figure 3:**
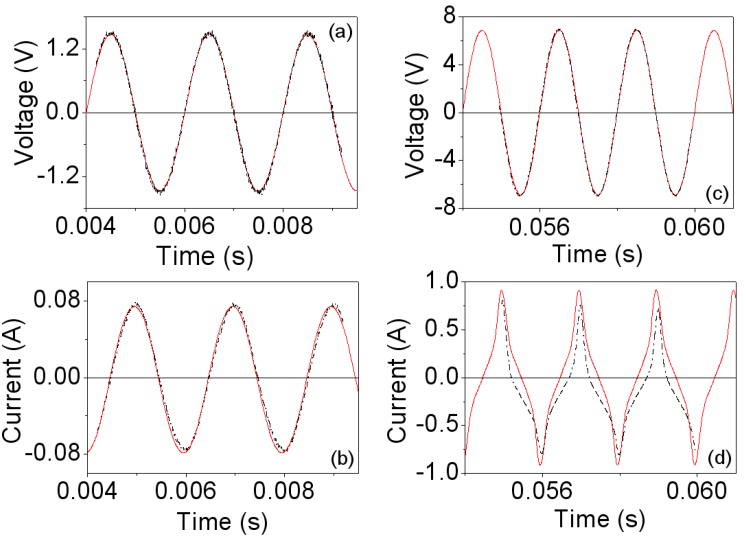
Experimental (-∙-∙-) and 2D simulated waveforms (—) at 500 Hz. (**a**,**b**) *V*_p_ ≈ 1.5 V and time step τ = 10^−4^ s; and (**c**,**d**) *V*_p_ ≈ 7.5 V and time step τ = 10^−4^ s.

The computed values of the Steinmetz coefficients are in this case *C*_m_ ≈ 10.23, α ≈ 1.5 and β ≈ 2.28. In Figure 3 we plot the voltage and current waveforms, obtained by experiment and by 2D simulations at 500 Hz. *V*_p_ is the amplitude of the voltage waveform. Figure 3a,b correspond to the linear region while Figure 3c,d are the results for saturation. As can be seen in Figure 3a,b, in the linear region the measured and simulated voltage and current waveforms are very similar. At high intensity, the experimental and simulated waveforms are not sinusoidal due to the saturation effect and remain similar showing good agreement between the results. The discrepancies between the experimental and simulated current waveforms shown in Figure 3d could be due to effects related to the construction of the inductor and to the determination of the Steinmetz coefficients. These coefficients have been computed from measurements taken using an RM14/I core. The RM14/I core geometry does not have a uniform cross sectional area throughout its path length, leading to local areas within the core that were not at the planned flux density level. These variances in flux density could explain the discrepancies.

**Figure 4:**
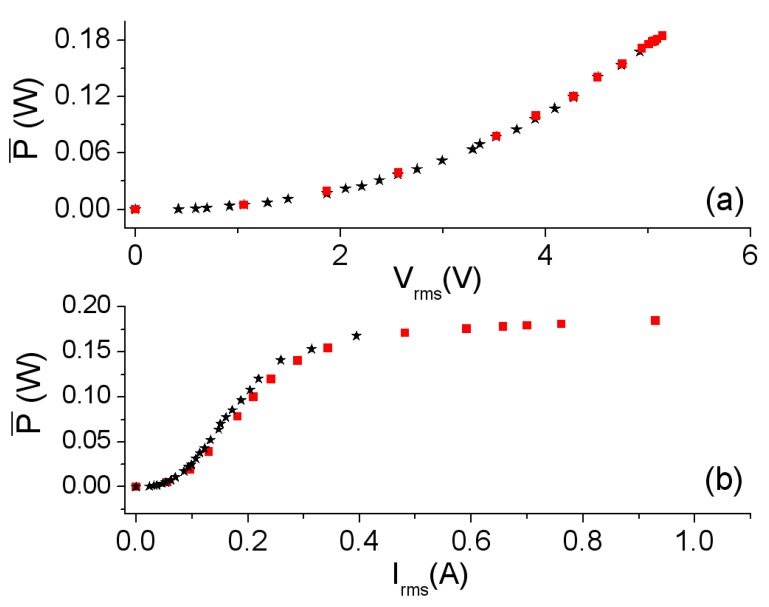
Results at 500 Hz. (**a**) $\bar{P}$-*V*_rms_ curve; and (**b**) $\bar{P}$-*I*_rms_ curve. Experiment (–*–) and 2D simulation (–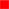
–).

**Figure 5:**
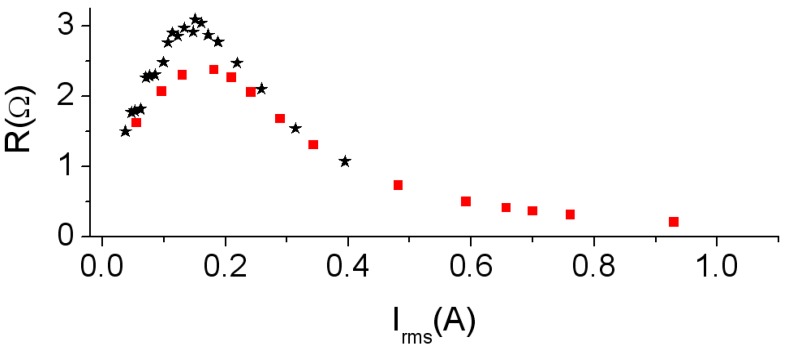
Results at 500 Hz. *R*-*I*_rms_ curve. Experiment (–*–) and 2D simulation (–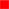
–).

Figure 4 shows the power $\bar{P}$ as a function of *V*_rms_ and the current *I*_rms_. The agreement between the experimental and simulated power curves (Figure 4a,b) is good. The design of the new 2D inductor model is capable of reproducing the nonlinear behavior of power and the effective current value in all working regions (linear, intermediate and saturation). $\bar{P}$ increases as the values of voltage *V*_rms_ and current *I*_rms_ increase showing an asymptotic behavior of the $\bar{P}$-*I*_rms_ curve towards the value $\bar{P}$ ≈ 0.2 W. This is due to the fact that an increase in the value of the voltage provokes an increment in the current that flows through the inductor. As a consequence Ф and *B* increase. Therefore, the generated hysteresis loop and power losses in the core also increase. In Figure 5 we show the *R*-*I*_rms_ curve (*R* as a function of *I*_rms_), which has been computed using the equation $R=\bar{P}/I_{\mathrm{rms}}^{2}.$ The shape of the experimental and 2D simulated *R*-*I*_rms_ curves are similar. Again, a nonlinear relation between *R* and *I*_rms_ can be observed. As *I*_rms_ increases, the values of *R* first reach a maximum of 2.5 Ω at 0.18 A and then decrease asymptotically towards a very small value.

### 3.2. Results at 40 kHz (Working Frequency of the Ferrite)

We obtain the Steinmetz coefficients from experimental measurements using the inductor excited by a sinusoidal voltage at 40 kHz. These have turned out to be: *C*_m_ ≈ 21.26, α ≈ 1.44 and β ≈ 3.

**Figure 6:**
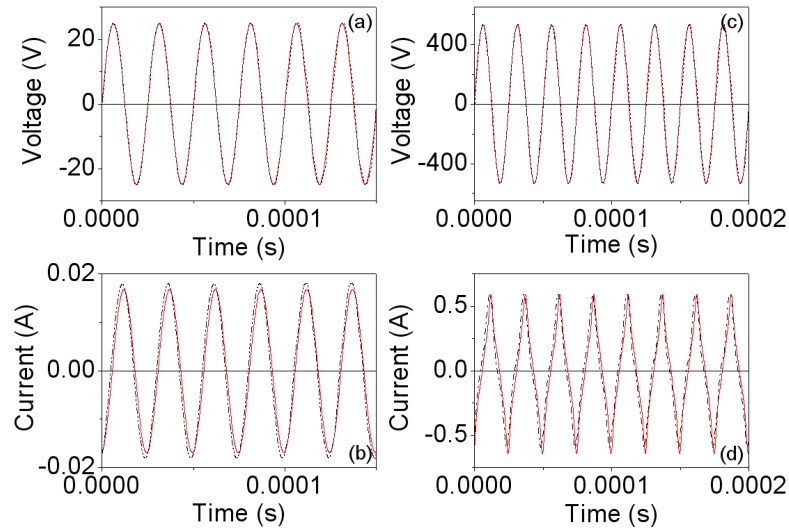
Experimental (-∙-∙-) and 2D simulated waveforms (—)at 40 kHz. (**a**,**b**) *V*_p_ ≈ 25 V and time step τ = 10^−6^ s; and (**c**,**d**) *V*_p_ ≈ 535 V and time step τ = 10^−6^ s.

**Figure 7:**
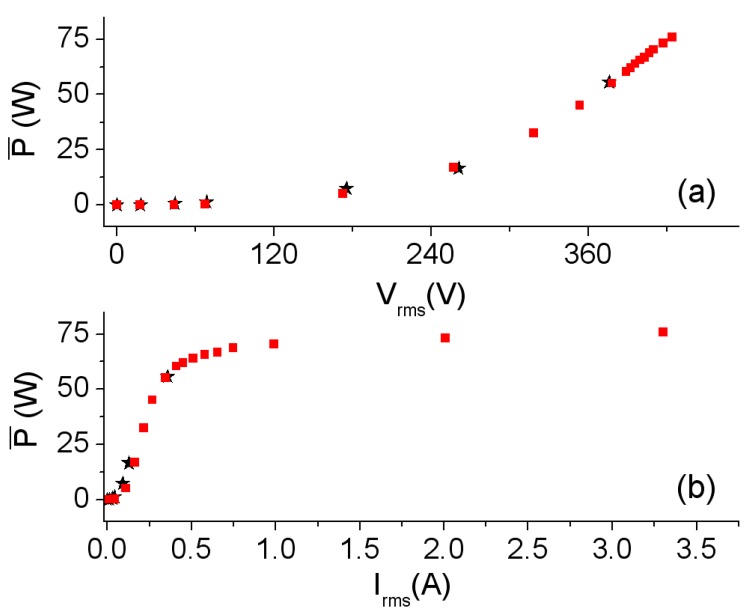
Results at 40 kHz. (**a**) $\bar{P}$-*V*rms curve; and (**b**) $\bar{P}$-*I*_rms_ curve. Experiment (–*–) and 2D simulation (–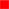
–).

In Figure 6 we show the waveforms obtained by experiment and by simulation at 40 kHz, where the effects of saturation can be seen in the deformation of the current waveforms. Similarly to the frequency of 500 Hz, we obtain good agreement between the waveforms. Figure 7a,b show the corresponding powers. The shape of the curves is similar to those corresponding to the frequency of 500 Hz, with nonlinear behavior existing between the power and voltage as well as between the power and current. Again, the power shows an asymptotic behavior at 80 W as *I*_rms_ increases.

**Figure 8:**
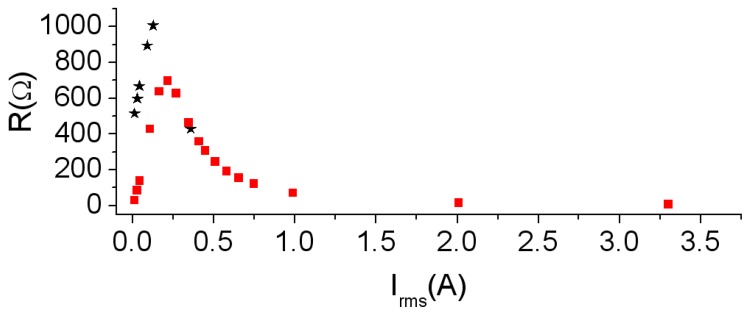
Results at 40 kHz. *R*-*I*_rms_ curve. Experiment (–*–) and 2D simulation (–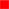
–).

In Figure 8 we plot the *R*-*I*_rms_ curve. The shape of the curves is similar as described previously at 500 Hz and the maximum is reached at 725 Ω for 0.25 A.

## 4. Discussion and Conclusions

In this paper we have proposed a specific modeling procedure for computing the power loss resistance of the equivalent electrical circuit of an inductor with an RM-type ferrite core. The procedure has been developed using finite element analysis in 2D and allows the transient simulation in 2D of the voltage, current, and power waveforms in the linear, intermediate and saturation regions at various working frequencies. We have used as software input parameters the experimental *B*-*H* curve that characterizes the core material and the Steinmetz coefficients measured for each frequency.

The modeling procedure involves three steps: *premodeling*, *simulation* and *postmodeling*, and the *R*-*I*_rms_ curve is derived at the *postmodeling step*. The effectiveness of the procedure has been analyzed for the case of a soft ferrite inductor (RM14/I core with 28 turns) under sinusoidal excitation at frequencies of 500 Hz (low frequency) and 40 kHz (working frequency of the ferrite), obtaining the following conclusions:

The new 2D inductor design for the RM/I type ferrite core is capable of reproducing the experimental shapes of the voltage and current waveforms from the linear to the nonlinear saturation regions and in the range of 500 Hz to 40 kHz. For all frequencies, the current waveforms are sinusoidal and contain harmonics when the ferrite reaches saturation. Apart from this, the new 2D design is capable of reproducing the nonlinear behavior of $\bar{P}$ with *I*_rms_, $\bar{P}$ with *V*_rms_, and *R* with *I*_rms_ in all working regions (linear, intermediate, and saturation) at the studied frequencies. $\bar{P}$ increases monotonically with *V*_rms_ and *I*_rms_ showing an asymptotic behavior of the $\bar{P}$-*I*_rms_ curve. The *R*-*I*_rms_ curve reaches a maximum and tends to a very small final value with increasing *I*_rms_. This happens both for the simulated and the experimental curves.

Finally, we have studied the influence of the frequency on the 28-turn RM14/I inductor at 500 Hz and 40 kHz, concluding that for both values of frequency the $\bar{P}$-*V*_rms_, $\bar{P}$-*I*_rms_ and *R*-*I*_rms_ curves have the same shape and for a fixed voltage, if the frequency increases, the current that flows through the winding decreases so that the generated hysteresis loops are increasingly smaller, reducing the power losses. Both $\bar{P}$ and *R* depend strongly on the frequency, obtaining for $\bar{P}$ in the saturation region values of 0.2 W at 500 Hz and 80 W at 40 kHz.

As future work, we propose carrying out a specific procedure to obtain the equivalent loss resistance under square excitation (typical of a power converter) with the aim of generating the equivalent electrical *L*-*R* circuit of the inductor, *L* being the inductor’s nonlinear inductance.
